# Advances in modeling gastric intestinal metaplasia: a comprehensive review of experimental models and mechanistic insights

**DOI:** 10.3389/fonc.2025.1664298

**Published:** 2025-11-19

**Authors:** Xinyuan Liu, Kaiqi Yang, Nan Zhang, Xiujing Sun

**Affiliations:** 1 Department of Gastroenterology, Beijing Friendship Hospital, Capital Medical University, Beijing, China; 2 State Key Laboratory of Digestive Health, Beijing, China; 3 National Clinical Research Center for Digestive Diseases, Beijing, China; 4 Beijing Digestive Disease Center, Beijing, China; 5 Beijing Key Laboratory for Precancerous Lesion of Digestive Diseases, Beijing, China

**Keywords:** gastric intestinal metaplasia, animal models, *Helicobacter pylori*, bile acid, gastric organoids

## Abstract

Epithelial cells within the gastric corpus mucosa exhibit a dynamic response to injury, characterized by alterations in gene transcription, cellular phenotype, and tissue organization, collectively termed metaplasia. Among these changes, gastric intestinal metaplasia (GIM) represents one of the most prevalent precancerous lesions associated with intestinal-type gastric cancer (GC). This pathological progression typically evolves through a sequence of stages: chronic atrophic gastritis, intestinal metaplasia, atypical hyperplasia, and ultimately, GC. A deeper understanding of GIM is crucial for advancing diagnostic and therapeutic strategies in GC management. Despite its clinical significance, progress in elucidating the underlying mechanisms of GIM has been limited, primarily due to the lack of reliable and reproducible animal models that accurately recapitulate this condition. This review systematically examines the existing mouse, rat, and organoid models utilized for GIM research, providing critical insights into various methodological approaches and potential mechanisms. Specifically, we investigate five pivotal aspects of pyloric metaplasia and GIM: *Helicobacter pylori* infection, bile acid induction, chemical agent interventions, transgenic technologies, and gastric organoids. Through this comprehensive analysis, we aim to establish a robust foundation for future research initiatives focused on unraveling the molecular mechanisms driving GIM development and formulating effective prevention and treatment strategies.

## 1 Introduction

Gastric intestinal metaplasia (GIM) is a well-established precancerous lesion or condition associated with a significantly elevated risk of gastric cancer (GC) (1). According to the classical Correa cascade theory, intestinal-type GC develops through a well-defined sequence of histopathological stages: from healthy gastric mucosa to chronic superficial gastritis, atrophic gastritis, GIM, dysplasia, and finally intestinal-type GC (2). GIM is classified into two distinct categories: complete and incomplete GIM. Complete intestinal metaplasia is marked by the presence of metaplastic glands composed of goblet cells, columnar cells with a well-defined brush border, and occasionally Paneth cells. This category is further subdivided into three subtypes: small intestine metaplasia type I, small intestine metaplasia type II, and colonic metaplasia (3). Subsequently, several reports indicate that GIM can also exhibit a mixed type combining both colonic and small intestinal phenotypes (4, 5). In contrast, incomplete GIM shows poorly formed brush borders, irregularly distributed immature goblet cells, and glands with predominantly colonic morphology. This subtype may also contain columnar cells with mixed phenotypic features, such as the presence of mucin droplets typically seen in goblet cells (6, 7). In essence, GIM involves the replacement of gastric epithelium with intestinal-type cells. As a result, proteins normally expressed in the intestine—including caudal type homeobox 2 (CDX2), mucin 2 (MUC2), and trefoil factor 3 (TFF3)—serve as useful molecular biomarkers for detecting and investigating GIM (7, 8).

When gastric corpus glands lose parietal and chief cells, they acquire morphological and molecular features resembling those of pyloric glands. This phenotypic shift is particularly noticeable at the base of atrophic corpus glands, where structural remodeling leads to the marked upregulation of trefoil factor 2 (TFF2) and other specific genes, conferring an antral gland-like appearance. The identification of a previously unrecognized basal cell lineage in these atrophic glands represents a definitive metaplastic transition, now classified as spasmolytic polypeptide-expressing metaplasia (SPEM) (9). Based on immunohistochemical profiling of markers such as Ki67 and Muc2, Goldenring et al. suggested that SPEM may progressively evolve into a GIM phenotype, indicating that GIM represents only one of several possible precursor or intermediate stages in gastric carcinogenesis (10). SPEM is considered a response in the gastric mucosal injury repair process and may also represent an early-stage lesion in gastric carcinogenesis. Nowadays, SPEM serves as a critical model for investigating the mechanisms underlying gastric carcinogenesis, particularly in elucidating the transition from chronic inflammation to cancer.

**Figure 1:**
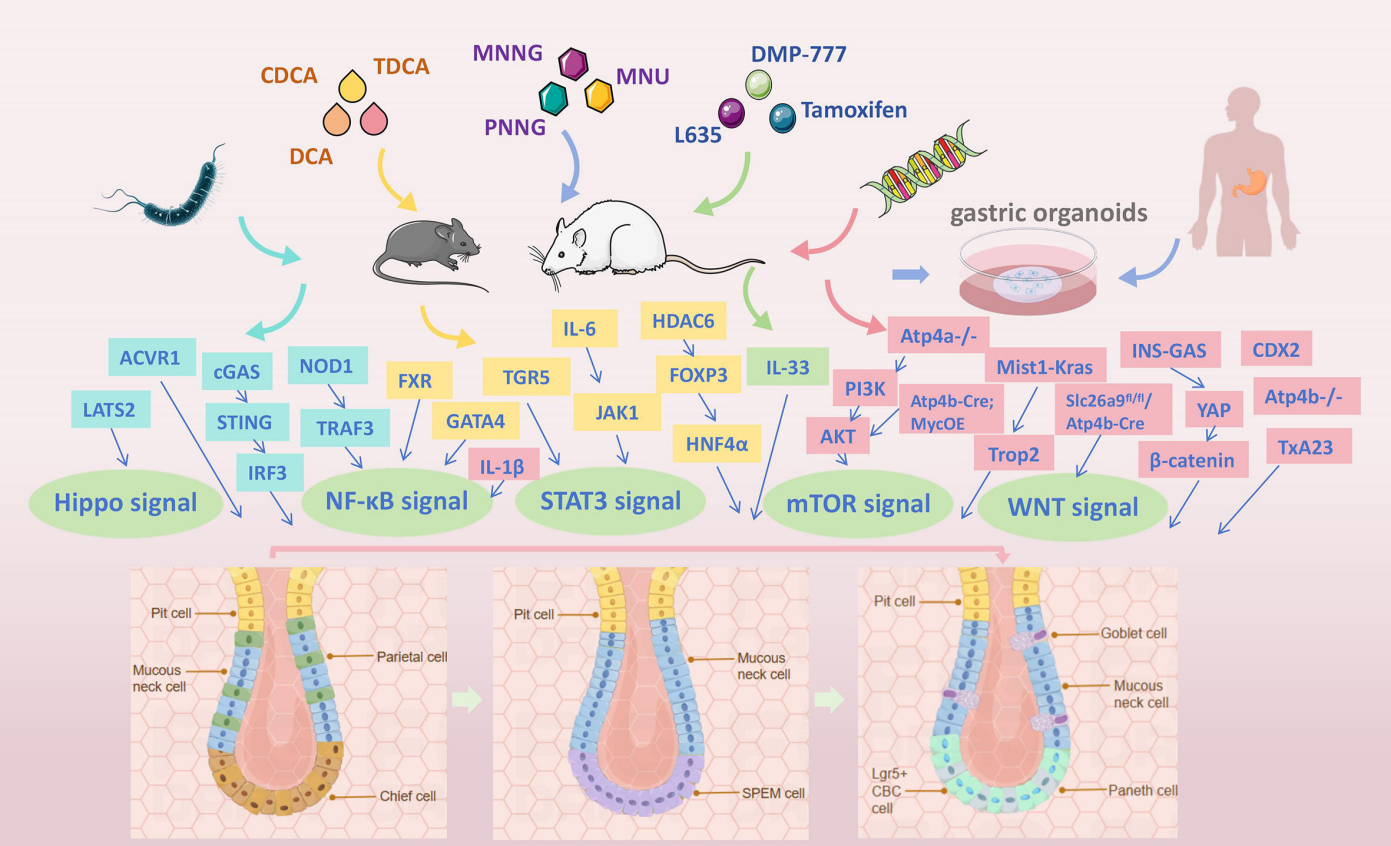
Overview of the GIM models, with particular emphasis on five key approaches: *H. pylori* infection, bile acid induction, chemical intervention, transgenic technologies, and gastric organoids.

The early detection and management of gastric precancerous lesions, particularly GIM, are vital to preventing GC and reducing its incidence. This necessitates a deeper investigation into the molecular mechanisms and signaling pathways governing the transition from normal mucosa to cancer. It also demands the development of physiologically relevant animal models that accurately simulate human disease for improved translational research. This review provides a comprehensive and systematic analysis of current methodologies for establishing GIM models, with particular emphasis on five key approaches: *Helicobacter pylori (H. pylori)* infection, bile acid induction, chemical agent intervention, transgenic technologies, and gastric organoids (**Figure 1**). By critically evaluating existing experimental evidence, we aim to establish a robust foundation for animal-based research on gastric precancerous lesions and to provide valuable theoretical insights that may guide future mechanistic studies and therapeutic development.

## 2 Experimental models of *H. pylori*-induced GIM

*H. pylori*, classified as a Class I carcinogen by the World Health Organization, plays a pivotal role in the development of gastritis, GIM, and GC (11). Given its strong association with gastric pathologies, *H. pylori* is widely used in experimental models to study precancerous lesions of gastric cancer (PLGC) and GC. In this section, we focus on summarizing the roles and mechanisms by which *H. pylori* contributes to the induction of GIM (**Table 1**). Chen et al. demonstrated that *H. pylori* infection upregulates the oncogene Activin A receptor type I (ACVR1), which subsequently promotes GIM by regulating CDX2 expression through comprehensive *in vivo* and *in vitro* experiments (12). A recent study further revealed that *H. pylori* infection activates the cGAS/STING/IRF3 signaling pathway, stimulating the kynurenine pathway of tryptophan metabolism. This metabolic shift leads to increased production of xanthurenic acid, a key mediator that drives the development of GIM (13). Additionally, *H. pylori* infection involves the Hippo LATS2/YAP1/TEAD signaling pathway, which is crucial for maintaining mucosal homeostasis. The Hippo signaling pathway acts as a protective mechanism to preserve the epithelial phenotype of gastric cells and limit *H. pylori*-induced precancerous lesions (14). Another study demonstrated that *H. felis*-infected mice developed characteristic gastric histopathological changes, including severe chronic gastritis with lymphoid follicle formation, mucous neck cell hyperplasia, oxyntic atrophy, and SPEM, with predominant pathological manifestations observed in the gastric corpus. Importantly, the study further revealed that both *H. felis*-induced SPEM and intestinal metaplastic phenotypes were reversible following bacterial eradication (15). Early studies showed that NOD1-deficient mice exhibit increased susceptibility to *H. pylori* infection (16). When NOD1-deficient mice were infected with *H. pylori* for 12 months, goblet cells were observed in the gastric mucosa, accompanied by significantly elevated expression of GIM markers (17). The GIM modeling process relying solely on *H. pylori* infection proves to be both time-intensive and technically challenging. To address these limitations, researchers have developed comprehensive modeling approaches that integrate *H. pylori* with additional modalities. For example, the Mist1-Kras mouse model, featuring tamoxifen-induced activation of the constitutively active Kras allele (G12D) in chief cells, effectively recapitulates the sequential progression from normal gastric epithelium to SPEM, GIM, and dysplasia. Valerie et al. further expanded the utility of this model by incorporating *H. pylori* infection. Their findings revealed that in *H. pylori*-infected Mist1-Kras mice, the staining patterns of griffonia simplicifolia lectin II (GS-II) and MUC2 exhibited consistent trends, characterized by a decrease in GS-II and an increase in MUC2 between 6 and 12 weeks post-infection (18). Moreover, using the same model, they demonstrated that *H. pylori* isolates collected from different disease stages of the same individual displayed distinct colonization capacities in both healthy and metaplastic gastric glands (19). This finding reveals that *H. pylori* dynamically evolves during gastric carcinogenesis—early-stage strains may preferentially colonize healthy mucosa, while late-stage strains adapted to metaplastic niches acquire a competitive advantage in lesioned tissues. Such colonization selectivity likely serves as a core driver of pathological progression. Numerous alternative *H. pylori* co-molding methodologies will be systematically discussed in subsequent sections. Critically, this pathogen drives GIM by orchestrating complex interactions between bacterial infection, host signaling pathways, and gastric microenvironmental remodeling, collectively advancing the development of gastric precancerous lesions.

**Table 1:** Animal models of *H. pylori* and bile acid-induced GIM.

Animals	Species	Types of *H. pylori* or Bile acids	Method	Duration	Targets and pathways	Markers	Phenotype	Reference
Mouse	C57BL/6	SS1	Via gavage (2×10^9^ CFU)/mouse	1 month	ACVR1	CDX2, MUC2, Villin-1	GIM	(12)
Mouse	C57BL/6	PMSS1	Via gavage (2×10^9^ CFU)/mouse	2 weeks	cGAS/STING/IRF3	CDX2, MUC2, Villin	GIM	(13)
Mouse	C57BL/6J	HPARE*	Via gavage	3–12 months	Hippo LATS2/YAP1/TEAD	CDX2, MUC2	GIM, dysplasia	(14)
Mouse	C57BL/6J	*H. felis* (ATCC 49179)	Via gavage (2.5 ×10^6^ CFU)/mouse	3, 6, 12 months	/	TFF2, MUC2, CD44, DCLK1, Villin	SPEM, GIM	(15)
Mouse	NOD1-deficient C57BL/6	ATCC 43504	Via gavage (2.5 ×10^8^ CFU)/mouse	12 months	NOD1/TRAF3/NF-κB	CDX2, MUC2, TFF3	GIM	(17)
Mouse	Mist1-Kras**	PMSS1	Via gavage (5 × 10^7^ CFU)/mouse	12 weeks	/	MUC2, TFF3	SPEM, GIM	(18, 19)
Mouse	C57BL/6J	DCA, CDCA	Via a plastic feeding tube (10 mM, 150 ul, bid)	45 days	FXR/NF-κB	CDX2, MUC2	GIM	(24)
Mouse	C57BL/6J	CDCA	Via a plastic feeding tube (10 mM, bid)	50 days	GATA4/NF-κB	CDX2, KLF4, MUC2	GIM	(25)
Mouse	C57BL/6J	TDCA	Via a feeding tube (120 mg/kg/d)	43 weeks	IL-6/JAK1/STAT3	/	gastritis	(26)
Mouse	C57BL/6J	/	Bile acid reflux surgery	50 Weeks	STAT3	/	dysplasia	(26)
Mouse	Rosa26^Hnf4α^	DCA	Adding to drinking water (0.3%)	12 months	HDAC6/FOXP3/HNF4α	KLF4, MUC2, CDX2	GIM	(28)
Mouse	INS-GAS	DCA	Adding to drinking water (0.2%)	6 months	TGR5/p-STAT3/KLF5	KLF5	GIM	(29)

*CagA and VacA positive.

**Mist1-CreERT2 Tg/+; LSL-K-Ras(G12D) Tg/+.

Emerging evidence from next-generation sequencing has uncovered a complex gastric microbial ecosystem, challenging the long-held notion of *H. pylori* as the exclusive etiological microorganism in gastric precancerous lesions and GC. A recent study demonstrated that gastric microbiota from GIM or GC patients exhibits selective colonization in murine stomachs, triggering precancerous lesions. Notably, histopathological analysis documented significant marked dysplastic changes by 12 months post-inoculation (20). This discovery provides novel insights into the mechanistic and translational modeling of GIM, while offering the prospect of intercepting gastric precancerous lesion progression through an innovative ecotherapeutic paradigm—precision microbiota modulation.

## 3 Experimental models of bile acid-induced GIM

Bile acids, which are cholesterol derivatives, play a vital role in fat absorption and transport and are predominantly found in organs such as the liver, gallbladder, and intestines (21). Hepatocytes synthesize these compounds through CYP-mediated cholesterol oxidation via two principal pathways. The classical pathway produces primary bile acids via cholesterol hydroxylase activity, including cholic acid (CA) and chenodeoxycholic acid (CDCA), which are subsequently conjugated to taurine (mice) or glycine (humans), forming TCA, TCDCA, GCA, and GCDCA (22). These are exported into the bile ducts via bile salt export pumps (23).

Bile acids can induce GIM through a variety of pathways and regulatory mechanisms in animal models (**Table 1**). In one study, researchers administered CDCA and DCA to C57BL/6J mice via a plastic feeding tube and discovered that bile acids could promote the upregulation of CDX2 and MUC2 in gastric epithelium via stimulating FXR/NF-κB signaling pathway (24). However, this study only validated molecular biomarkers of GIM without corresponding histopathological evidence, likely because hematoxylin and eosin (HE) staining and alcian blue-periodic acid schiff (AB-PAS) staining did not detect significant goblet cell changes. Using a similar modeling approach, Yang et al. demonstrated that CDCA promotes GATA4 expression via NF-κB signaling, while GATA4 and CDX2 form a positive feedback loop that synergistically enhances MUC2 transcription in GIM (25). Taurodeoxycholic acid (TDCA) is significantly and positively correlated with the lipopolysaccharide-producing bacteria in the gastric juice of bile reflux gastritis and GC patients. Then the researchers employed two distinct modeling approaches: TDCA tube feeding and bile acid reflux (BR) surgery in mice. Gastric inflammation was induced after 45 weeks of TDCA administration, and histopathological analysis revealed that 3 out of 8 mice in the BR surgical group developed gastric lesions, including one precancerous lesion and two cases of atypical hyperplasia (26).

In addition to studies conducted on C57 wild-type mice, researchers have also utilized mice with specific genetic backgrounds for bile acid intervention modeling. Wang et al. developed a transgenic mouse model in which Lgr5+ gastric mucosal stem cells specifically expressed Hnf4α. This was achieved by crossing Lgr5-Cre and LSL-Hnf4α mice, both on a C57BL6 background, to activate Hnf4α expression (27). Building on this model, Zhang et al. constructed Rosa26Hnf4α transgenic mice, which were administered bile acids (0.3% DCA, pH 7.0) in the drinking water for 12 months. Their findings demonstrated that HNF4α overexpression, combined with DCA treatment, induced the gastric mucosa to secrete intestinal mucus and led to abnormal mucosal structures, including enlarged glands at the squamocolumnar junction and gastric mucosal atrophy (28). Additionally, transgenic INS-GAS mice on an FVB/N genetic background were chronically exposed to 0.2% DCA dissolved in their drinking water for a period of 6 months. Researchers observed that DCA administration significantly increased serum total bile acid levels and accelerated the sequential development of GIM and subsequent dysplasia (29). INS-GAS mice, characterized by pancreatic islets secreting carboxyamidated gastrin-17, exhibited elevated serum amidated gastrin levels, marked thickening of the oxyntic mucosa, and an increased bromodeoxyuridine (BrdU) labeling index in the gastric body (30). These mice eventually developed progressive parietal cell depletion and hypochlorhydria, spontaneously progressing to GIM, dysplasia, and GC by 20 months of age (31). Undoubtedly, using INS-GAS mice for bile acid feeding can significantly shorten the time required to establish GIM models while increasing the success rate. Taken together, these studies highlight the critical role of bile acids in driving gastric mucosal changes and the progression of precancerous lesions. They also underscore the importance of utilizing diverse mouse models, including genetically engineered strains, to better understand the molecular mechanisms underlying GIM and its transition to GC. These findings provide valuable insights for developing targeted interventions to prevent or treat gastric precancerous conditions.

## 4 Experimental models of MNNG-induced GIM

Methyl-N’-nitro-N-nitrosoguanidine (MNNG), a potent environmental chemical carcinogen, is strongly associated with the development of PLGC and GC (32). MNNG was instrumental in establishing the first rat model for gastric tumors, although the underlying mechanisms remain largely unexplored (33). Subsequently, MNNG was widely used in the modeling of gastric precancerous lesions (**Table 2**). Wistar rats treated with MNNG (83 mg/mL) in drinking water for ≥4 months exhibited GIM in 80-100% of cases (34). Lower concentrations (25, 50, 100 mg/mL) in drinking water over 32 weeks also consistently induced GIM (35). Drinking MNNG solution (200 μg/mL) combined with alternating hunger-satiety cycles effectively induced PLGC in Sprague Dawley (SD) rats (36–38). To better replicate complex pathogenesis, models combining MNNG (170 μg/mL) with feeding schedules like 1-day feed/1-day fast or 2-day feed/1-day fast cause gastric mucosal thinning, gland reduction, and GIM (39). Additionally, GIM was observed during the construction of a chronic atrophic gastritis model in rats fed MNNG for 12 weeks (40).

**Table 2:** Animal models of chemical carcinogen-induced GIM.

Animals	Species	Chemicals	Dosages	Methods	Duration	Phenotype	Reference
Rat	Wistar	MNNG	83 ug/ml	Adding to drinking water	4 months	GIM	(34)
Rat	Wistar	MNNG	25, 50, 100 ug/ml	Adding to drinking water	32 weeks	GIM	(35)
Rat	SD	MNNG	200 ug/ml	Adding to drinking water	20 weeks	GIM	(36)
Rat	SD	MNNG	200 ug/ml	Adding to drinking water	16 weeks	GIM	(37, 38)
Rat	SD	MNNG	170 ug/ml	Adding to drinking water	10 weeks	GIM	(39)
Rat	Wistar	MNNG	167 ug/ml	Adding to drinking water	12 weeks	GIM	(40)
Rat	SD	MNNG*	170 ug/ml	Adding to drinking water	12 weeks	GIM	(41)
Rat	SD	MNNG*	200 ug/ml	Adding to drinking water	40 weeks	GIM	(42)
Rat	Wistar	MNNG*	100 ug/ml	Adding to drinking water	16 weeks	GIM	(43)
Rat	Wistar	MNNG*	120 ug/ml	Adding to drinking water	32 weeks	GPL	(44)
Rat	SD	MNNG	170 ug/ml	By oral gavage	24 weeks	GIM	(45)
Rat	Wistar	MNNG	0.02 mol/L	By oral gavage	24 weeks	GIM	(46)
Rat	Wistar	MNNG*	200 mg/kg & 600μg/kg	By oral gavage	days 0 and 14, 32 weeks	GIM	(47)
Rat	SD	MNNG**	200 ug/ml	By oral gavage	8 weeks	GIM	(48)
Rat	SD	MNNG**	200 ug/ml	By oral gavage	20 weeks	GIM	(49)
Rat	SD	MNNG	5 ml/kg & 200 ug/ml	By oral gavage & Adding to drinking water	20 weeks	GIM	(50)
Rat	Wistar	PNNG	59.5 mg/ml	Adding to drinking water	4, 8, and 12 months	GIM	(51)
Mouse	BABL/C	MNU	300μg/ml	Adding to drinking water	20 weeks	GIM	(54)
Meriones unguiculatus	Mongolian gerbils	MNU +HP (ATCC 43504)	20 ppm & 30 ppm	Adding to drinking water	20 weeks	GIM	(56)
Meriones unguiculatus	Mongolian gerbils	MNU +HP(ATCC 43504)	10 ppm	Adding to drinking water	20 weeks	GIM	(57)
Mouse	C57BL/6	MNU +HP (SS1)	240 ppm	Adding to drinking water	5 weeks	GIM	(58)
Rat	Wistar	MNU +X-ray irradiation	100 ppm	Adding to drinking water	15 weeks	GIM	(53)

*MNNG is not the only medication used in model construction.

**The rats were also given an ammonia solution with a concentration of 0.1%.

In studies utilizing MNNG to establish animal models of GIM, specific chemical co-factors are often required to more effectively simulate the complex environment of human GIM development and enhance modeling efficiency. These co-factors operate through distinct mechanisms, synergizing with MNNG to promote gastric mucosal damage, inflammation, and metaplastic transformation. Key chemical synergistic agents commonly employed in research include ranitidine, sodium salicylate, ethanol, and high-salt diet. Ranitidine, an H2 receptor antagonist that alters the gastric microenvironment by suppressing gastric acid secretion, was incorporated into feed (0.03%, 0.05%, 0.3%) to enhance lesion development (41–44). Sodium salicylate which induce mucosal injury and inflammation was administered via gavage (2% solution at 10 mL/kg) on fasting days (41). This study revealed that Celastrus orbiculatus ethyl acetate extract could reverse PLGC progression by modulating the PDCD4-ATG5 signaling pathway. Ethanol was directly used to compromise the gastric mucosal barrier and trigger inflammation and mucosal damage (40% ethanol at 10 mL/kg/d via gavage) (42). Integrated protocols combining MNNG (120-200 μg/mL in water), ranitidine feed, specific fasting regimens, and gavage of irritants like ethanol, sodium salicylate, or high salt have successfully established robust PLGC and GIM models for evaluating therapeutic interventions.

Compared to adding MNNG to drinking water, administering MNNG to mice via gastric gavage is a more direct and effective approach. For instance, SD rats were given an MNNG solution dissolved in drinking water containing 5% alcohol by gavage every two days for 24 weeks. This model was used to investigate the potential mechanisms of GIM and found that OLFM4 contributes to the progression of GIM through activation of the MYH9/GSK3β/β-catenin pathway (45). Similarly, Wistar rats were delivered 0.02 mol/L MNNG solution by gavage for 24 weeks, in combination with a hunger-satiety disorder regimen, and subjected to emotional stress via the tail-pinching method weekly to establish PLGC animal models (46). Rats were given MNNG at 200 mg/kg by oral gavage on days 0 and 14, followed by saturated NaCl (1 ml per rat) three times per week for the first three weeks. Subsequently, MNNG (600 µg/kg) and saturated NaCl were administered every other day. Following 35 weeks of induction, moderate to severe GIM was observed in the gastric antrum of model rats (47). Lv et al. utilized the MNNG–ammonia composite modeling method to establish a rat model of PLGC, demonstrating that Ginsenoside Rg3 induced apoptosis and inhibited proliferation in rats with PLGC (48). Using the same modeling approach, Zeng et al. found that GRg3 attenuated angiogenesis and moderated microvascular abnormalities in rats with PLGC, potentially through its suppression of the aberrant activation of GLUT1 and GLUT4 (49). Another PLGC rat model was established by administering MNNG at 5 ml/kg via gavage once a week, combined with free access to MNNG solution (200 μg/ml) in drinking water. This model was used to investigate the effects and mechanisms of Atractylenolide III on PLGC (50). In summary, combining MNNG with other factors (dietary disruption, chemical irritants, stress) significantly enhances model fidelity by better mimicking the multifactorial etiology of human gastric precancerous lesions.

N-Propyl-N’-nitro-N-nitrosoguanidine (PNNG), a propyl derivative of MNNG, is also a potent nitroso compound carcinogen used to induce gastric lesions. Administered to Wistar rats via drinking water (59.5 μg/ml), PNNG induced GIM in the glandular stomach with increasing incidence over time: 25% at 4 months, 75% at 8 months, and 83% at 12 months (51). Although PNNG has been shown to have a weaker carcinogenic effect on the stomach compared to MNNG, GIM was observed in the glandular stomach following exposure to PNNG (52).

## 5 Experimental models of MNU-induced GIM

N-methyl-N-nitrosourea (MNU), a potent direct-acting carcinogen, induces tumors in multiple organs and contributes to gastric carcinogenesis, including the development of GIM (53). Key modeling approaches and findings are shown in **Table 2**. Mice administered 300 μg/mL MNU in drinking water combined with alternate-day fasting for 20 weeks developed GIM. AB-PAS staining confirmed the presence of acidic mucin characteristic of GIM (54). Combining MNU in drinking water with *H. pylori* inoculation consistently induces both preneoplastic and neoplastic gastric lesions in murine models (55). Rats inoculated with *H. pylori* and treated with MNU exhibited higher degrees of inflammation, GIM, and submucosal multicystic glands compared to those inoculated with *H. pylori* alone (56). Long-term *H. pylori* infection combined with MNU exposure results in more severe inflammatory cell infiltration, hyperplasia, GIM, and cellular proliferation (BrdU labeling) in the gastric mucosa than shorter infection durations with MNU (57).

A specific mouse model using cyclic MNU administration (1 week on/1 week off for 5 weeks) plus totally three times of *H. pylori* inoculation resulted in either preneoplastic or neoplastic lesions in 9 out of 19 mice: 4 with GIM, 1 with dysplasia, and 4 with adenocarcinoma (58). An early study noted X-ray irradiation increased GIM and alkaline phosphatase (ALP)-positive foci. While MNU effectively drives carcinogenesis and GIM formation, particularly with *H. pylori* co-exposure, the study found it did not significantly accelerate the progression of existing GIM to gastric tumors in rats (53). MNU is a significant tool for modeling gastric carcinogenesis and GIM. Its effects are markedly potentiated by concurrent *H. pylori* infection, leading to robust and consistent development of preneoplastic lesions in rodent models.

## 6 Experimental models of chemical agent-induced SPEM

Beyond MNNG and MNU, specific chemicals are used to induce metaplasia, particularly acute models of SPEM in mice (**Table 3**). The transition to SPEM in murine gastric injury models is driven by the synergistic action of parietal cell disappearance and microenvironmental immune cell cytokine signals (59). Tamoxifen, DMP-777, and L635 are prominent agents for inducing acute SPEM. Huh et al. demonstrated that tamoxifen injection (5 mg/20g mouse, 3 consecutive days) causes massive parietal cell loss (>90%), hyperproliferation of stem/progenitor cells, and chief cell morphological changes (60). Saenz et al. elucidated a tamoxifen administration regimen to recapitulate oxyntic atrophy and the early preneoplastic events leading to gastric dysplasia (61). Both tamoxifen and DMP-777 can induce metaplasia even after prior parietal cell ablation (62). Miao et al. used high-dose tamoxifen/DMP-777 models with scRNA-seq to show that metabolic and mitochondrial changes are critical for damage response, regeneration, and metaplasia (paligenosis) (63).

**Table 3:** Animal models of chemical agent-induced SPEM.

Animals	Species	Chemicals	Dosages	Methods	Duration	Phenotype	Reference
Mouse	C57BL/6	DMP-777	350 mg/kg	Oral	7 days or 14 days	SPEM	(62, 63)
		Tamoxifen	5 mg/20 g	Intraperitoneal injections	3 days		
Mouse	C57BL/6	DMP-777	350 mg/kg	Oral	7 days or 14 days	SPEM	(64)
		L635	350 mg/kg	Oral	3 days		
		Tamoxifen	5 mg/20 g	Intraperitoneal injections	3 days		
Mouse	C57BL/6	Tamoxifen	5 mg/20 g	Intraperitoneal injections	3 days	SPEM	(65, 66)
Mouse	C57BL/6	5-fluorouracil + Tamoxifen	150 mg/kg, bid + 250 mg/kg	Intraperitoneal injections	2 days + 3 days	SPEM	(67)
Mouse	BALB/c	Tamoxifen	5 mg/20 g	Intraperitoneal injections	3 days	SPEM	(68)

Wfdc2-knockout mice are resistant to oxyntic atrophy, SPEM, and M2 macrophage accumulation in models induced by DMP-777, L635, or tamoxifen. Exogenous WFDC2 protein upregulates IL-33, promotes M2 macrophage differentiation, and drives SPEM pathogenesis (64). These acute drug-induced models using tamoxifen or DMP-777 are crucial tools to study *H. pylori* interactions with metaplastic tissues, evaluate therapeutic agents, and dissect the fundamental cellular and molecular mechanisms driving SPEM (65–67). Kevin A. et al. conducted single-cell RNA sequencing in both tamoxifen-induced parietal cell ablation and chronic (4-month) TxA23 autoimmune gastric metaplasia mouse models. Their findings revealed that SPEM development follows a conserved cellular program regardless of etiology, while acquiring immunoregulatory properties specifically in chronic inflammatory contexts (68). These models consistently highlight the indispensable roles of parietal cell disappearance and specific immune signaling, particularly involving M2 macrophages and cytokines like IL-33 in SPEM pathogenesis. They reveal a core conserved cellular program for SPEM development while also uncovering context-specific features, significantly advancing the understanding of gastric metaplasia mechanisms.

## 7 GIM transgenic animal models

Transgenic mice serve as essential models for investigating gene function, elucidating disease mechanisms, and facilitating drug development, significantly advancing the field of biomedical research. Petersen et al. summarized transgenic mouse models of parietal cell loss, including Claudin-18 null mice, Kruppel-like factor 4 null mice, Runx3 null mice, and H/K-cholera toxin mice. These models could start with normal mucosa and then develop progressively increasing levels of atrophy and metaplasia (69). In this section, we summarize the transgenic mouse models utilized in the study of GIM (**Table 4**). The Atp4a gene encodes a subunit of the H+/K+-ATPase proton pump essential for gastric acid production. Knocking out Atp4a impairs parietal cell function, leading to chronic achlorhydria and hypergastrinemia, triggering progressive precancerous changes in aged mice, including hyperplasia, mucolytic alterations, and GIM (70, 71). Atp4a knockout mice recapitulate human GIM development linked to acid loss (72–75). Studies using Atp4a-/- mice have revealed activation of the Warburg effect and PI3K/AKT/mTOR signaling during GIM progression and the potential of metformin to inhibit GIM by suppressing inflammation and apoptosis pathways (73, 74). The protective effects of traditional formulations like Weiwei decoction exerted protective effects against SPEM in Atp4a-/- mice (75). Thus, Atp4a knockout mice provide a powerful platform for dissecting GIM mechanisms and evaluating therapeutic interventions.

**Table 4:** GIM transgenic animal models.

Animals	Genetic modification	Species	Duration	Markers	Phenotype	Reference
Rat	Atp4a^-/-^	a mixed 129SvJ and Black Swiss	10–12 weeks	/	GIM	(70)
Mouse	Atp4a^-/-^	a mixed 129SvJ and Black Swiss	12 months	/	Incomplete GIM	(72)
Mouse	Atp4a^-/-^	C57BL/6	16 weeks	MUC2	GIM	(73)
Mouse	Atp4a^-/-^	/	24 weeks	CDX2	GIM	(74)
Mouse	Atp4a^-/-^	/	10 weeks	Clu, Cftr, Wfdc2, Dmbt1, Gpx2, GSII*, Clusterin	SPEM	(75)
Mouse	Atp4b^-/-^	BALB/c	12 months	TFF2, Clu, MUC6, CD44, WFDC2 (HE4)	SPEM	(76)
Mouse	Slc26a9^fl/fl^/Atp4b-Cre	C57BL/6J	6 months; 14 months	TFF2, MUC6;CDX2, MUC2, TFF3	SPEM, GIM, Dysplasia	(77)
Mouse	Atp4b-Cre; *Myc*^OE^	C57BL/6	25 weeks; 35 weeks	MUC2	GIM, Adenoma	(78)
Mouse	K-ras	C57BL/6	3 months	/	GIM, Dysplasia	(79)
Mouse	Mist1-Kras** (Tamoxifen)	/	3–4 months	Clu, GSII, CD44v9, TFF3, MUC2, CDX1, CDX2, Trop2	SPEM, GIM, Dysplasia	(80–82)
Mouse	IL-1β IL-1β + HP (ATCC 49179)	C57BL/6	12 months	/	GIM, Dysplasia	(83)
Mouse	IL-1β + *H. felis*	C57BL/6	12 months	/	GIM, Dysplasia	(84)
Mouse	INS-GAS + *H. felis*	FVB/N	6 weeks***	/	GIM	(85)
Mouse	INS-GAS + HP (PMSS1)	C57BL/6	4 months***	/	GIM	(86)
Mouse	INS-GAS + HP (PMSS1)	/	16weeks***	CDX2	GIM	(87)
Mouse	CDX2	/	1–15 months	Villin, FABP1, MUC2, TFF3, GCC	GIM	(88)
Mouse	CDX2	C57BL/6	37 days, 244 days	MUC6, MUC5AC	GIM	(89)
Mouse	TxA23	BALB/c	4 months	TFF2, WFDC2 (HE4)	SPEM	(90)

*Griffonia simplicifolia (GSII) lectin binds MUC6.

**Mist1-CreERT2 Tg/+; LSL-K-Ras(G12D) Tg/+.

***Duration of *H. pylori* infection.

Similarly, ATPase H+/K+ transporting subunit beta (Atp4b) deficient mouse models have been used to study the relationship between achlorhydria and hypergastrinemia. Compared to Atp4a-/- mice, Atp4b-/- mice exhibited more severe hyperplasia and cyst formation but fewer focal changes such as adenomas, polyps, and submucosal invasion by GC, revealing significant distinctions in gastric pathological manifestations between these experimental models (71, 76). Notably, Atp4b knockout mice have been shown to develop SPEM (76). In an innovative study, parietal cell-specific Slc26a9 knockout mice (Slc26a9fl/fl/Atp4b-Cre) were generated by crossing Slc26a9fl/fl with Atp4b-Cre mice. This model sequentially developed parietal cell loss and oxyntic atrophy with mucous metaplasia at 1 and 6 months, progressing to high-grade intraepithelial neoplasia (HGIN) by 14 months (77). Additionally, researchers observed varying degrees of GIM in Atp4b-Cre; MycOE mice at 12, 25, and 35 weeks of age, with the most severe GIM occurring at 25 weeks (78).

Studies have also explored the role of oncogenic mutations in gastric metaplasia. For example, the presence of oncogenic K-ras mutations in K19-expressing gastric epithelial progenitor cells triggers inflammatory pathways, leading to gastric atrophy, metaplasia, and dysplasia (79). In a tamoxifen-induced genetic engineering model, the expression of activated Ras in the chief cells of Mist1-Kras mice induced a complete spectrum of metaplastic changes, including both SPEM and GIM (80). This genetically engineered mouse model has significantly shortened the time required for modeling GIM, representing a discovery of considerable importance. Consistent with these findings, two independent studies demonstrated that Mist1-Kras mice developed distinct pathological features over time: pyloric metaplasia glands containing SPEM cell lineages were observed as early as 1 month post-induction, followed by the progression to dysplastic glands at 4 months post-induction (81, 82).

Another study found that over 70% of aged transgenic mice with high-expression polymorphisms of interleukin-1β (IL-1β) exhibited severe hyperplasia, chronic inflammation, tissue atrophy, metaplasia, and dysplasia. When these IL-1β transgenic mice were infected with H. felis, they developed exacerbated gastric inflammation and more pronounced histopathological changes within five months post-infection (83). Interestingly, H+/K+-ATPase-IFN-γ mice crossed with IL-1β transgenic mice did not exhibit spontaneous gastric epithelial hyperplasia or mucosal heterotopia. However, IL-1β transgenic mice infected with H. felis showed rapid progression of metaplasia and high-grade dysplasia (84).

The INS-GAS transgenic mouse model, which causes hypergastrinemia, has been previously mentioned in the context of bile acid-induced GIM (29). This model has been extensively used to study gastric carcinogenesis, although it undergoes a distinct GIM phase before progressing to GC. Following *H. pylori* infection, INS-GAS mice develop GIM as early as 6 weeks post-infection, characterized by elongation of epithelial columnar cells, formation of microvillous brush borders, and the appearance of cytoplasmic lipid vesicles resembling goblet cells (85). Four months post-infection, male INS-GAS mice exhibited significant gastric mucosal pathology, including pronounced inflammatory cell infiltration and marked GIM. Notably, GC progression in this model shows sexual dimorphism, with aged male INS-GAS mice developing spontaneous lesions more rapidly, a process accelerated by *H. pylori* infection (86). However, not all *H. pylori*-infected INS-GAS mice developed GC, although they exhibited significant inflammation and mild GIM (87).

CDX2, an intestine-specific transcription factor, is minimally expressed in normal gastric mucosa. Its ectopic expression is strongly associated with the development of GIM, making CDX2 a crucial molecular marker for this condition (8). Foxa3/Cdx2 transgenic mice, which ectopically express CDX2, develop GIM with goblet cells restricted to the distal stomach (88). Similarly, Mutoh et al. generated a CDX2 transgenic mouse model that exhibited complete disruption of normal mucosal architecture by postnatal day 37, characterized by extensive goblet cell distribution and columnar intestinal-type epithelial cells with well-developed microvilli. This metaplastic transformation persisted throughout the experimental period, as evidenced by the stomachs of transgenic mice on day 244, which were entirely replaced by intestinal metaplastic mucosa (89). By 4 months of age, TxA23 mice exhibit autoimmune-driven chronic gastritis characterized by parietal cell atrophy, hyperplasia of mucous neck cells, and the development of SPEM. This model has proven instrumental in dissecting the contributions of immune cell subsets and cytokine networks to the pathogenesis of gastritis and gastric metaplasia (90). Based on the TxA23 mouse model, Christine et al. generated TxA23×Il4rα mice, which develop gastritis but lack expression of the IL-4/IL-13 receptor subunit IL-4Rα. The study demonstrated that IL-13 promotes metaplastic epithelial changes associated with gastric carcinogenesis (91). These studies collectively highlight the utility of genetic models in understanding the mechanisms of gastric metaplasia and underscore the critical roles of parietal cell dysfunction, inflammatory signaling, and biomarkers of GIM like CDX2 in the development of GIM and SPEM.

## 8 Gastric organoids and GIM

Gastric organoids, renowned for recapitulating normal and tumor tissue characteristics, are revolutionizing gastric disease research. Jin et al. established organoids from FVB/N and INS-GAS mice, using *in vitro* deoxycholic acid (DCA) treatment to elucidate the TGR5/p-STAT3/KLF5 signaling axis in gastric epithelium (29). Advances now enable generation from mouse corpus and antrum, forming 2-D epithelial monolayers after 6–7 days of 3D culture, providing robust models. Human gastric organoids were also generated from sleeve gastrectomy biopsies. Co-culture studies with *H. pylori* and dendritic cells using these monolayers demonstrated induction of TLR9 expression, IFNα secretion, and Schlafen-expressing Myeloid-Derived Suppressor Cell (SLFN-MDSC) polarization; crucially, *in vivo* IFNα neutralization attenuated *H. pylori*-induced SPEM development (92). Researchers established a temporal progression model using Mist1-Kras mice, isolating gastric glands 3- and 4-months post-tamoxifen for organoid culture (Meta3/Meta4). Meta4 organoids displayed significant architectural abnormalities, including multilayered organization, mirroring *in vivo* glandular hyperplasia, and basal fission at 4 months (93). Furthermore, novel mouse organoids stably expressing HNF4A or CDX2 were developed. Both factors activated intestinal differentiation markers (alkaline phosphatase, lysozyme). Using CDX2 enhancer-deficient organoids, researchers proved that HNF4A-mediated intestinalization depends on CDX2 signaling, revealing mechanisms of gastric epithelial plasticity (94). Wataru et al. generated organoids from the antral region of mice following *H. pylori* infection or MNU carcinogen treatment (240 ppm) (15). Transplantation into immunodeficient NOD/SCID mice revealed MNU-derived organoids exhibited accelerated proliferation, abnormal morphology, and formed tumors within two months. Similarly, Li et al. induced GIM in C57BL/6J mouse organoids using MNNG, showing RAS pathway activation drives GIM progression, recapitulating human gastric GIM molecular and histopathological features (95). A recent study employed human GIM-derived organoids to demonstrate that nitazoxanide effectively attenuates CagA-induced SPEM (96). Sarah et al. performed multi-omics analysis on human normal gastric, GIM, and colon/ileum organoids, capturing genetic/epigenetic perturbations in GIM and establishing a unique progression model (97). These organoids represent valuable cellular models that reflect the underlying biological mechanisms of the GIM process. In summary, gastric organoids have emerged as a versatile and powerful tool in gastric disease research, bridging the gap between *in vitro* and *in vivo* studies. Their ability to recapitulate complex biological processes and disease states makes them invaluable for advancing our understanding of gastric biology and developing targeted therapeutic strategies.

## 9 Conclusion and perspectives

As GIM gains recognition as a clinically significant precancerous condition, there is an urgent need to establish a stratified framework integrating molecular drivers with clinicopathological features to optimize risk-adapted surveillance protocols. A synthesis of evidence from existing models—including *H. pylori* infection, bile acid exposure, chemical carcinogens, and genetically engineered systems—reveals several convergent mechanisms driving GIM pathogenesis. Chronic inflammation, triggered by *H. pylori* or chemical agents, initiates a cascade of epithelial damage and reparative reprogramming. Parietal cell loss, induced pharmacologically or through inflammatory injury, disrupts normal glandular homeostasis and creates a permissive microenvironment for metaplastic transformation. This is frequently accompanied by aberrant activation of CDX2 and other intestinal transcription factors, which redirect gastric epithelial differentiation toward an intestinal phenotype. Together, these core pathways—chronic inflammation, parietal cell depletion, and CDX2-driven reprogramming—constitute a unifying framework for GIM development. Moving forward, integrated modeling strategies that combine genetic manipulation with complementary insults (e.g., *H. pylori*, bile acids, or dietary shifts) will enhance pathophysiological relevance and experimental efficiency. Furthermore, organoid models offer significant advantages for investigating the pathogenesis of GIM, including their remarkable ability to recapitulate the *in vivo* microenvironment, experimental tractability, and high reproducibility. However, conventional GIM organoids are primarily epithelial models. They lack critical components of the native tumor immune microenvironment (TIME), such as immune cells, cancer-associated fibroblasts, and the complex extracellular matrix. This limits their ability to fully replicate the disease biology and predict responses to therapies that involve the immune system. Introducing specific immune cells into traditional organoid systems for co-culture may effectively simulate the TIME, offering a highly promising research strategy for future studies. Despite the variety of methods for animal modeling, the field still lacks a standardized and reproducible scheme for establishing GIM models. This methodological limitation poses substantial challenges in investigating the precancerous stages of gastric carcinogenesis. Therefore, there is an urgent need to leverage advancements in genetic engineering and biotechnology to develop more reliable experimental models and deepen our understanding of GIM pathogenesis. In the future, leveraging multiple models and integrating multi-omics data will enable the construction of a comprehensive GIM profile and the elucidation of its heterogeneity. The application of machine learning (ML) and artificial intelligence technologies to analyze multi-omics data will further facilitate disease prediction, biomarker discovery, and the identification of novel drug targets.
